# Ethnic and gender specific life expectancies of the Singapore population, 1965 to 2009 – converging, or diverging?

**DOI:** 10.1186/1471-2458-13-1012

**Published:** 2013-10-26

**Authors:** Raymond Boon Tar Lim, Huili Zheng, Qian Yang, Alex Richard Cook, Kee Seng Chia, Wei Yen Lim

**Affiliations:** 1 Saw Swee Hock School of Public Health, National University of Singapore, MD3, 16 Medical Drive, Singapore 117597, Singapore

**Keywords:** Life expectancy, Mortality, Singapore, Gender differences, Ethnic differences, Converging, Diverging

## Abstract

**Background:**

The increase in life expectancy and the persistence of expectancy gaps between different social groups in the 20^th^ century are well-described in Western developed countries, but less well documented in the newly industrialised countries of Asia. Singapore, a multiethnic island-state, has undergone a demographic and epidemiologic transition concomitant with economic development. We evaluate secular trends and differences in life expectancy by ethnicity and gender in Singapore, from independence to the present.

**Methods:**

Period abridged life tables were constructed to derive the life expectancy of the Singapore population from 1965 to 2009 using data from the Department of Statistics and the Registry of Births and Deaths, Singapore.

**Results:**

All 3 of Singapore’s main ethnic groups, and both genders, experienced an increase in life expectancy at birth and at 65 years from 1965 to 2009, though at substantially different rates. Although there has been a convergence in life expectancy between Indians and Chinese, the (substantial) gap between Malays and the other two ethnic groups has remained. Females continued to have a higher life expectancy at birth and at 65 years than males throughout this period, with no evidence of convergence.

**Conclusions:**

Ethnic and gender differences in life expectancy persist in Singapore despite its rapid economic development. Targeted chronic disease prevention measures and health promotion activities focusing on people of Malay ethnicity and the male community may be needed to remedy this inequality.

## Background

Life expectancy—the average number of years a person would live if he or she experienced the age-specific mortality rates of a given country in a particular year—is one of the most widely used measures of the health status of populations [1]. Life expectancy at birth has generally increased worldwide since the early 20^th^ century, with the exception of Sub-Saharan Africa [2], where the high prevalence of Human Immunodeficiency Virus has, instead, lowered life expectancy [2]. The main determinants of this general increment include public health interventions to prevent communicable diseases, such as vaccination and antibiotics treatment [3,4], rising living standards, improvements in nutrition and sanitation, progress in healthcare and health services, access to quality health services, lifestyle changes and education [5,6]. Women—now—generally outlive men in developed countries although the magnitude of this gender gap varies considerably [7]. The gender gap in life expectancy has been converging in most developed countries since the late 20^th^ century [8-10], which has been attributed to decreasing cardiovascular and cancer mortality (particularly lung cancer) in males, as well as narrowing disparity in chronic disease risk factors like smoking [9,10].

In addition, differences in life expectancy between ethnic groups living in the same country are well-established in the West [11,12]. In the United States, for example, blacks in general have lower life expectancy at birth than whites, while Hispanics appear to have higher rate than either [11,12]. However, such data are sparse in Asia, and life expectancy differences in subgroups in Asian countries are not well-documented.

Disparities in life expectancy among ethnic groups and between genders within a country often imply the presence of health inequalities. Studies have found important determinants to explain this variation, such as differences in the prevalence of chronic disease risk factors, preventive health behaviour, access to and utilisation of healthcare service, as well as socio-economic status [13,14].

Long-term monitoring of life expectancy by subgroups within a country—such as ethnic groups and gender—will provide useful information that will allow policy makers and other stakeholders to allocate healthcare resources, and plan health promotion programmes more effectively.

From a British colony to an independent country after separation from Malaysia in 1965, Singapore has undergone rapid economic development. Social and health changes in life expectancy and fertility have resulted in a concomitant demographic transition. This South-east Asian multiethnic city-state with people of Chinese, Malay and Indian ethnicities now faces an increasingly ageing population [15]. Though Singapore’s 3 main races differ substantially in their rates of diseases such as cancer [16] and heart disease [16], their differential mortality and life expectancy have not been studied. In this study, we examine the secular trends in life expectancy among gender and ethnicity groups in Singapore from 1965 (when it separated from a union with Malaysia) to the present, a time period over which gross domestic product per capita (2010 US$) rose from ~ US$500 to ~ US$41,000 [17].

## Methods

Data on mortality from 1940 to 2010 were obtained from the National Registry of Births and Deaths, Singapore [18]. Under the Registration of Births and Deaths Act (enacted in 1937), it is compulsory to register all births and deaths in Singapore [19]. The National Registry is based on the death certificate issued by a doctor following the death of any person who dies in Singapore, regardless of residential status. The certificate states the underlying causes of death according to the International Classification of Diseases (ICD) [18]. Deaths—limited to the 3 main ethnicities, which constituted more than 96% of all deaths—were then classified according to the 3 major mortality categories in accordance with the Global Burden of Disease Study, World Health Organisation (WHO) [20]. Group I causes include mortality from communicable diseases, maternal conditions, perinatal conditions and nutritional deficiencies (ICD-9 codes 001–139, 260–269, 320–322, 460–487, 630–676, 760–779); Group II, mortality from all non-communicable diseases (all other ICD-9 codes not used for Group I and Group III causes); and Group III, mortality from road traffic accidents, intentional and unintentional injuries (ICD-9 codes E800 to E999) [20].

In addition, period abridged life tables that depicted the mortality experience of a given population were constructed to derive the life expectancy of the Singapore population from 1965 to 2009. Complete life tables cannot be compiled as population and mortality data for every single year of age were not available. 3 essential components were used to construct the abridged life tables: mid-year population in each age group, the number of deaths in each age group for each 5-year period and the number of live births over each 5-year period. The mid-year population was obtained from the Department of Statistics [21], while the number of live births and deaths were obtained from the Registry of Births and Deaths [18]. The methodology for constructing the abridged life tables for the Singapore population was adopted from previous work in the demographic literature [22-24] and all formulae used in the computation process are detailed in Appendix I. The statistical programming language R (version 2.14.0) was used to plot the figures [25].

Demographic information on gender and ethnicities were taken from the National Registry which also issues a birth certificate to each registered newborn in Singapore. “Chinese” is defined as a person of Chinese origin, who in Singapore is most commonly Hokkien, Teochew, Cantonese, Hainanese, Hakka or Foochow, including their descendants, all of whom are classified as Chinese [26]. “Malay” is defined as an individual whose ancestors are from the Malay Archipelago typically Muslim, speaking the Malay language (Bahasa Melayu) and adhering to Malay customs [26]. “Indian” in the Singapore context is defined as a person whose origins are in now Bangladesh, India, Pakistan or Sri Lanka, most commonly Tamils, Telugus, Malayalis, Punjabis and Bengalis [26]. Singaporeans of mixed parentage have traditionally been classified according to their father’s ethnicity group [18] with the exception of those of mixed European and Asian descent who were labeled during colonial times as “Eurasian” to distinguish them from “pure” Europeans [27].

We limit our calculation of the mid-year population to only the 3 major ethnic groups as they made up more than 96% of the total resident population; the other minority groups such as Eurasians are excluded due to small numbers (less than 4% of the total population). The life expectancy data are then stratified according to gender and ethnicity.

Data for these analyses were obtained from publicly available aggregate information published by the Department of Statistics and Registry of Births and Deaths. No individual-level information was used, and there was no contact with any human subjects. Ethical review was thus deemed unnecessary for this study.

## Results

### Epidemiological transition of Singapore

The mid-year resident population of Singapore has increased from 2.0 million in 1968 to 3.8 million in 2010, of which 74-78% were Chinese, 13-15% were Malays and 7-9% were Indians during this time period. The predominant group of causes of mortality in 1940 and 1950 was communicable diseases (Group I), which accounted for 57% of all deaths in 1940. This had declined to 37% in 1960 and 15% in 1980 (Table 1), and is indicative of the epidemiological transition that took place in Singapore starting from the post-World War Two period. By 1980, mortality from non-communicable diseases (Group II, 78%) far exceeded the mortality from communicable diseases (Group I, 15%). Non-communicable diseases (Group II) were the predominant causes of death between 1970 and 2010, representing 71% to 80% of all deaths. In addition, the total mortality rate per 100,000 resident population has undergone a gradual decline through the years, consistent with increasing life expectancy in Singapore.

**Table 1:** Epidemiological transition of Singapore from 1940 to 2010 classified according to the 3 major mortality categories of the global burden of disease study, WHO

**Year**	**Group I causes (%) mortality from communicable diseases, maternal conditions, perinatal conditions and nutritional deficiencies**	**Group II causes (%) mortality from non-communicable diseases**	**Group III causes (%) mortality from road traffic accidents, intentional and unintentional injuries**	**Total deaths**	**Total mortality per 100,000 resident population**	**Mid-year resident population (million)**
1940	8,974 (57)	6,274 (40)	457 (3)	15,705	-	-
1950	6,781 (55)	5,031 (41)	500 (4)	12,312	-	-
1960	3,802 (37)	5,967 (58)	517 (5)	10,286	629.46	2.0 [1968]
1970	2,258 (21)	7,623 (71)	836 (8)	10,717	516.61	2.0
1980	1,857 (15)	9,749 (78)	899 (7)	12,505	518.03	2.3
1990	1,827 (13)	11,056 (80)	1,008 (7)	13,891	513.51	2.7
2000	2,147 (14)	12,413 (79)	1,133 (7)	15,693	450.92	3.3
2010	3,092 (18)	13,545 (77)	973 (5)	17,610	436.84	3.8

### Gender-differences in life expectancy

**Figure 1:**
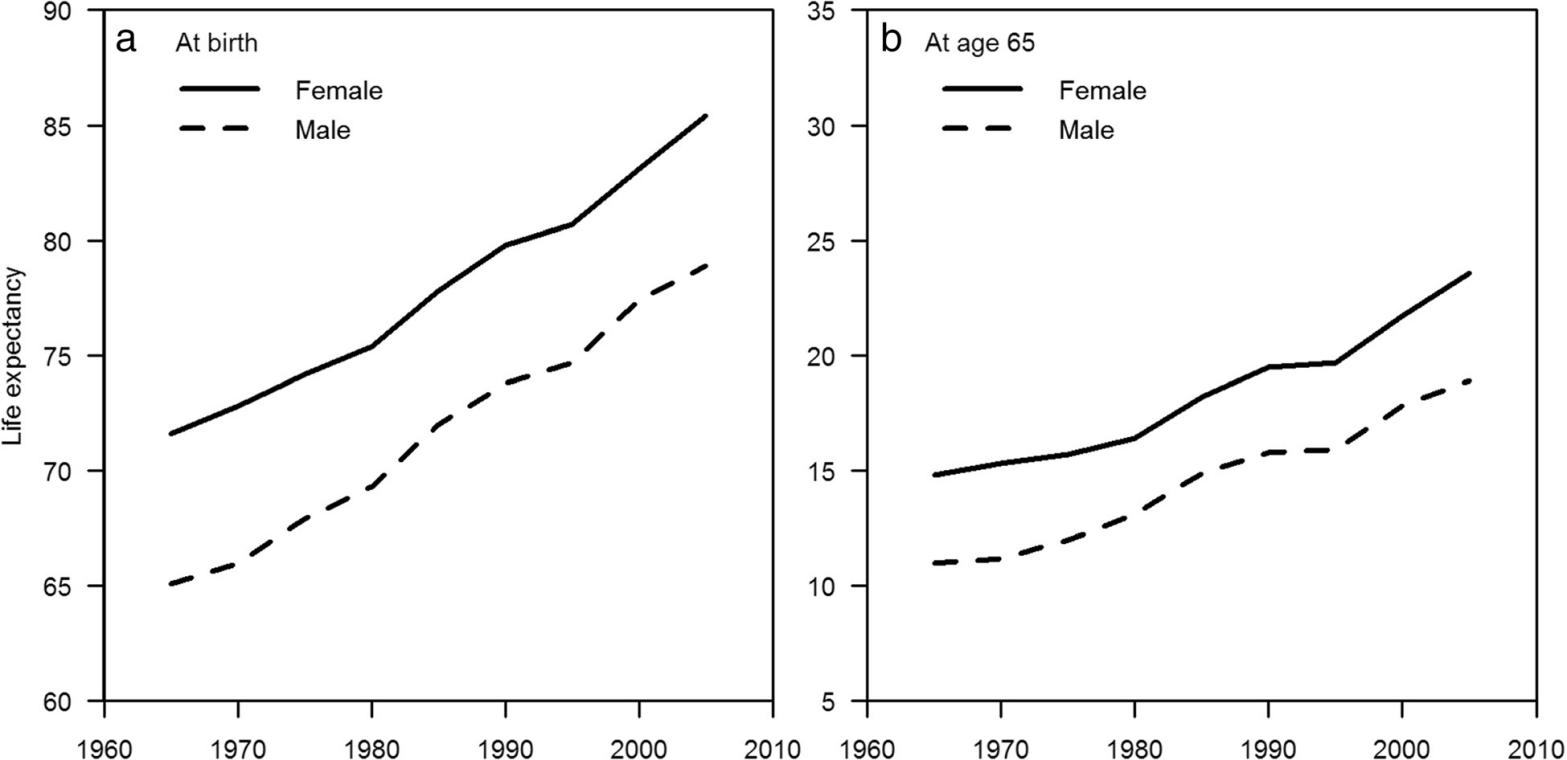
Life expectancy at birth (a) and at age 65 (b) for both genders, Singapore, from 1965 to 2009.

In the period from 1965 to 1969, females had a life expectancy at birth of 71.6 years, 6.5 years longer than males (65.1 years). The life expectancy at birth of both genders grew between 1965 and 2009: by 2005 to 2009, the life expectancy at birth of males was 78.9 years, while that for females was 85.4 years. Although the rate of increase in life expectancy was higher in males than females in the late 1970s and early 1980s, the rate of increase in females was greater from the late 1980s onwards (Figure 1a and Table 2). As a result, the gap in life expectancy between males and females (5–7 years throughout this period) has not narrowed (Figure 1a). Similar results were seen for life expectancy at 65 years, with overall secular improvements in life expectancy. In 1965, females at age 65 had a life expectancy of 14.8 years, while males had 11.0 years, a 3.8 years gap. Again, the rate of increase in life expectancy was higher in males than females in the late 1970s and early 1980s, but the rate of increase in females was greater from the late 1980s onwards (Figure 1b and Table 2). The gap in life expectancy at age 65 in 2005 was 4.7 years (Figure 1b), and a gap of about 3–5 years at age 65 has persisted from 1965 to 2009.

**Table 2:** Absolute and increase (relative to the five years prior) in life expectancy for both genders, Singapore, from 1965 to 2009

**Absolute life expectancy for both genders, Singapore, from 1965 to 2009**										
	**Year**	**1965**	**1970**	**1975**	**1980**	**1985**	**1990**	**1995**	**2000**	**2005**
At birth	Female	71.6	72.8	74.2	75.4	77.8	79.8	80.7	83.1	85.4
	Male	65.1	66.0	67.9	69.3	72.0	73.8	74.7	77.4	78.9
At age 65	Female	14.8	15.3	15.7	16.4	18.2	19.5	19.7	21.7	23.6
	Male	11.0	11.2	12.0	13.1	14.9	15.8	15.9	17.8	18.9
**Increase in life expectancy for both genders, Singapore (relative to the five years prior), from 1965 to 2009**										
	**Year**	**1965**	**1970**	**1975**	**1980**	**1985**	**1990**	**1995**	**2000**	**2005**
At birth	Female	-	1.2	1.4	1.2	2.4	2.0	0.9	2.4	2.3
	Male	-	0.9	1.9	1.4	2.7	1.8	0.9	2.7	1.5
At age 65	Female	-	0.5	0.4	0.7	1.8	1.3	0.2	2.0	1.9
	Male	-	0.2	0.8	1.1	1.8	0.9	0.1	1.9	1.1

**Figure 2:**
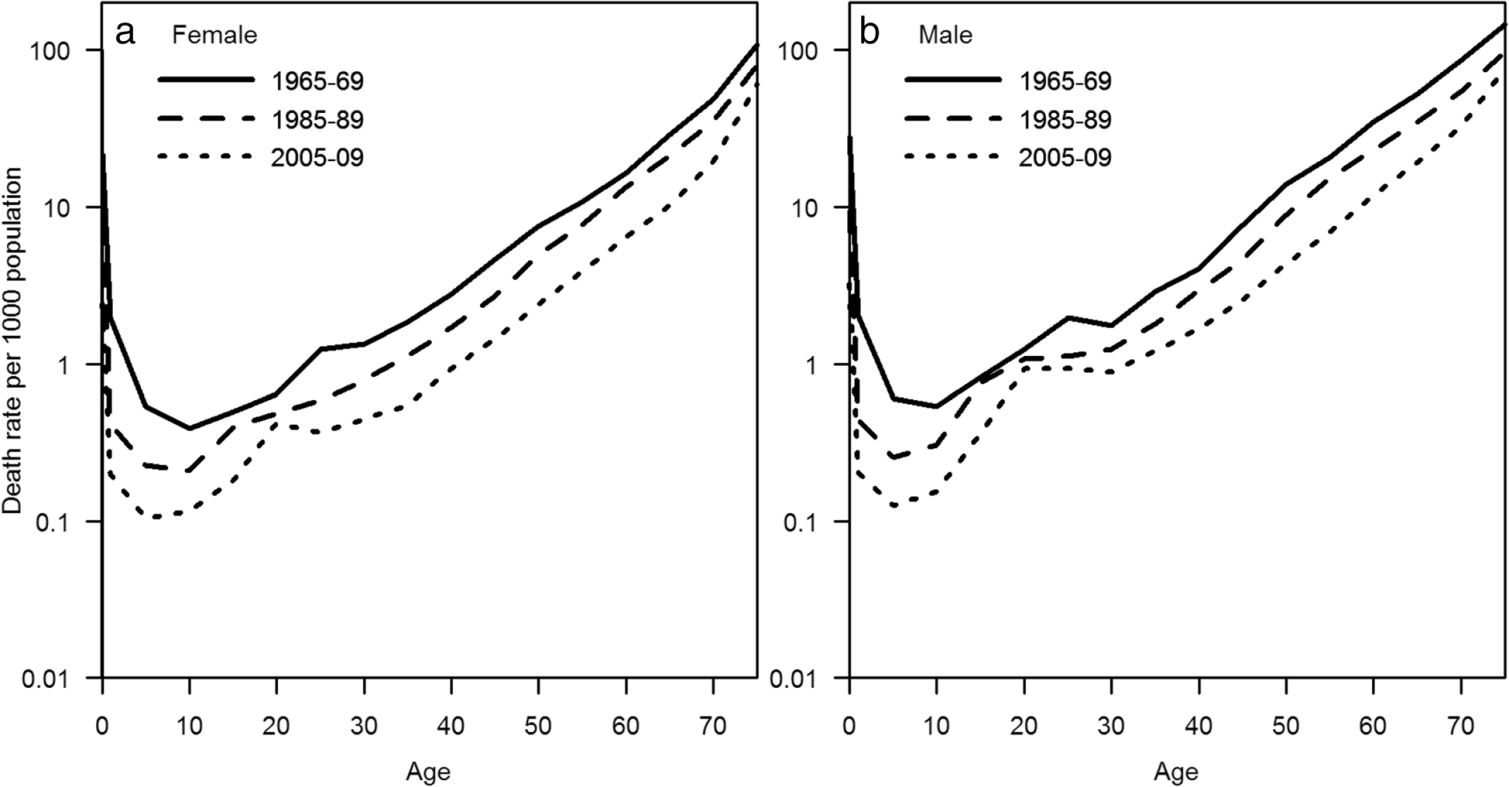
Age-specific mortality rates for, female (a) and male (b), Singapore, at three time points from 1965 to 2009 (semi-logarithmic scale).

Both genders experienced a decline in mortality rate during the period under observation. Mortality rate gaps between both genders were seen in 1965–1969, and have persisted. This gender gap is seen from childhood onwards, and persists throughout the life course for all 3 periods with females having a consistently larger decline in mortality rate (Figure 2).

### Comparison of life expectancy stratified by ethnicity in both genders

A Chinese girl born in 2005 can expect to live to 86.6 years. In contrast, a Malay boy can expect to live only to 74.8 years, a gap of nearly 12 years. Life expectancy for children of other ethnicity /gender combinations can expect a life expectancy between these extremes.

**Figure 3:**
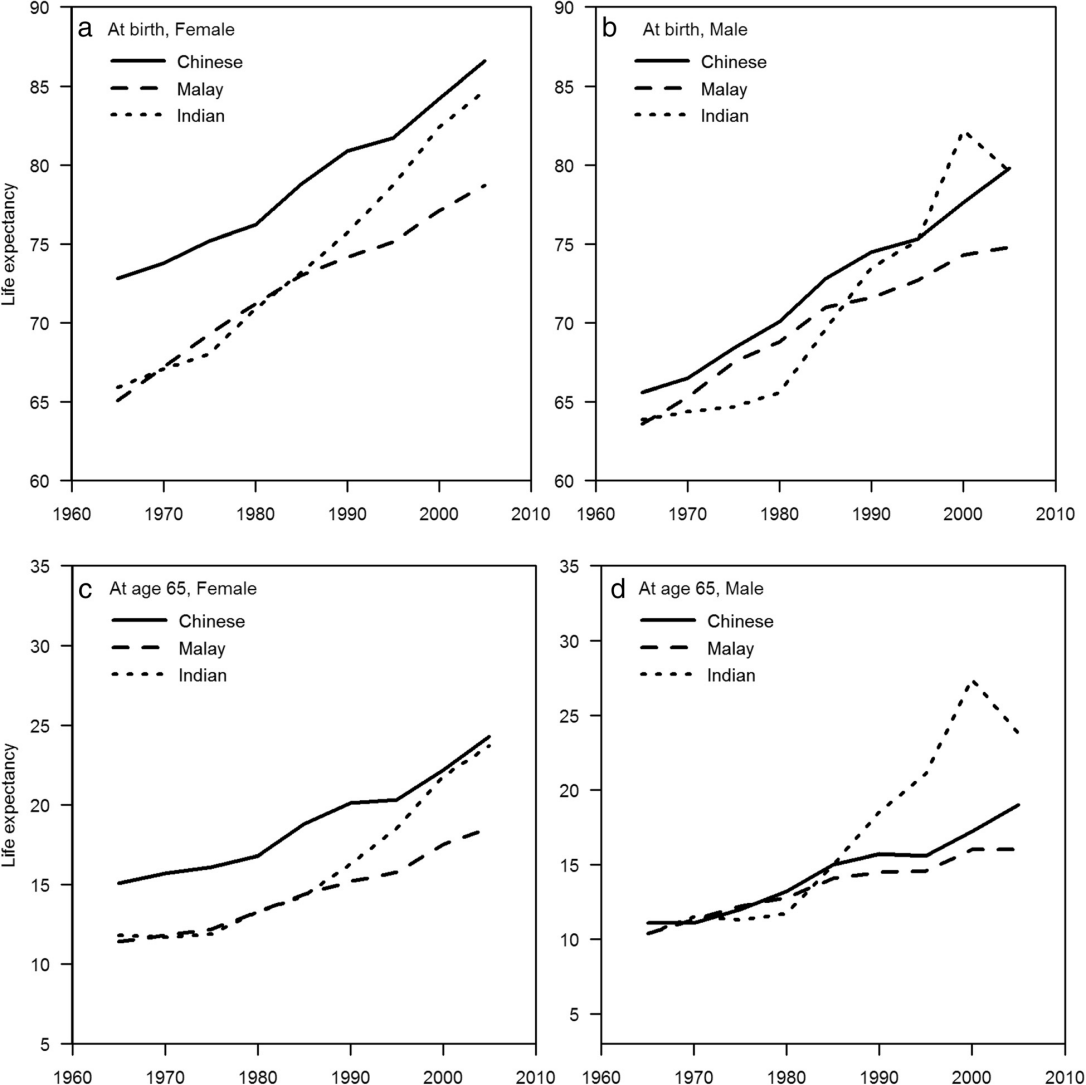
Life expectancy at birth (a & b) and at age 65 (c & d) by ethnicity and gender, Singapore, from 1965 to 2009.

The gap in life expectancy at birth between males and females persisted after stratifying by ethnicity, with a gap of about 5–7 years throughout this period. Further, although life expectancy for all 3 major ethnic groups has increased over the 50 years period, significant differences both in the magnitude of life expectancy and rate of increases in life expectancy are seen among ethnic groups. The greatest overall gains in life expectancy have been by the Indians, whose life expectancy at birth has increased by 18.8 years in females and 15.7 years in males, followed by the Chinese, where it has increased by 13.8 years in females and 14.2 years in males. The Malays, however, have seen an increase of only 13.6 years in females and 11.2 years in males. Despite the 5–7 years gap between Chinese males and females, Malay females have a life expectancy that is slightly lower than Chinese males from 1990 to 2009. A 2–4 years gap in the life expectancy at birth between Malay males and Malay females, as well as a persistent 8–11 years gap between Malay males and Chinese females existed throughout the period of observation from 1965 to 2009. The increase in life expectancy experienced by both Malay males and females exceeded that of Chinese in the 1970s, but this increase has since dropped off and the life expectancy at birth gap between the Chinese and Malays has widened in recent years (Figure 3 and Table 3). It is also striking that the gap in life expectancy at birth between Chinese and Malay males (of about 1–3 years, widening to 5 years in 2005) is much smaller than that between Chinese and Malay females (of about 5–8 years). Because the overall increases in life expectancy in these 4 groups are relatively similar, the gap between Chinese and Malays seen in 1965 has persisted, both in males and females.

**Table 3:** Absolute and increase (relative to the five years prior) in life expectancy by ethnicity and gender, Singapore, from 1965 to 2009

**Absolute life expectancy by ethnicity and gender, Singapore, from 1965 to 2009**										
	**Year**	**1965**	**1970**	**1975**	**1980**	**1985**	**1990**	**1995**	**2000**	**2005**
At birth	Chinese females	72.8	73.8	75.2	76.2	78.8	80.9	81.7	84.2	86.6
	Chinese males	65.6	66.5	68.4	70.1	72.8	74.5	75.3	77.6	79.8
	Malay females	65.1	67.2	69.3	71.2	73.0	74.2	75.1	77.1	78.7
	Malay males	63.6	65.3	67.5	68.8	71.0	71.6	72.7	74.3	74.8
	Indian females	65.9	67.1	68.0	70.9	73.2	75.7	78.7	82.4	84.7
	Indian males	63.9	64.4	64.7	65.6	69.6	73.5	75.2	82.2	79.6
At age 65	Chinese females	15.1	15.7	16.1	16.8	18.8	20.1	20.3	22.2	24.3
	Chinese males	11.1	11.1	12.0	13.2	15.0	15.7	15.6	17.2	19.0
	Malay females	11.4	11.8	12.2	13.3	14.4	15.2	15.8	17.5	18.5
	Malay males	10.4	11.3	12.2	12.8	14.1	14.5	14.6	16.0	16.0
	Indian females	11.8	11.7	11.9	13.3	14.3	16.3	18.5	21.8	23.7
	Indian males	10.1	11.5	11.3	11.7	15.0	18.5	21.1	27.4	23.8
**Increase in life expectancy by ethnicity and gender, Singapore (relative to the five years prior), from 1965 to 2009**										
	**Year**	**1965**	**1970**	**1975**	**1980**	**1985**	**1990**	**1995**	**2000**	**2005**
At birth	Chinese females	-	1.0	1.4	1.0	2.6	2.1	0.8	2.5	2.4
	Chinese males	-	0.9	1.9	1.7	2.7	1.7	0.8	2.3	2.2
	Malay females	-	2.1	2.1	1.9	1.8	1.2	0.9	2.0	1.6
	Malay males	-	1.7	2.2	1.3	2.2	0.6	1.1	1.6	0.5
	Indian females	-	1.2	0.9	2.9	2.3	2.5	3.0	3.7	2.3
	Indian males	-	0.5	0.3	0.9	4.0	3.9	1.7	7.0	-2.6
At age 65	Chinese females	-	0.6	0.4	0.7	2.0	1.3	0.2	1.9	2.1
	Chinese males	-	0.0	0.9	1.2	1.8	0.7	-0.1	1.6	1.8
	Malay females	-	0.4	0.4	1.1	1.1	0.8	0.6	1.7	1.0
	Malay males	-	0.9	0.9	0.6	1.3	0.4	0.1	1.4	0.0
	Indian females	-	-0.1	0.2	1.4	1.0	2.0	2.2	3.3	1.9
	Indian males	-	1.4	-0.2	0.4	3.3	3.5	2.6	6.3	-3.6

In contrast, although Indian males and females had the lowest life expectancy of the 3 major ethnicities in 1970s, Indian males and females have both experienced the highest rates of increases in life expectancy at birth, especially from 1990s onwards, such that the life expectancy gap has closed from 6.9 years between Chinese and Indian females and 1.7 years between Chinese and Indian males in 1965, to 1.9 years in females and 0.2 years in males (Figure 3 and Table 3).

These trends are mirrored for life expectancy at age 65, where a persistent gap is also seen between males and females, and between Malays and the other 2 races. Again, from the lowest life expectancy at age 65 of the 3 ethnic groups in the 1970s, Indian males and females have experienced rapid increases in life expectancy, and the gap between Chinese and Indians has substantially closed. In fact, since 1985, Indian males surviving to 65 outlive their Chinese peers on average (Figure 3 and Table 3).

**Figure 4:**
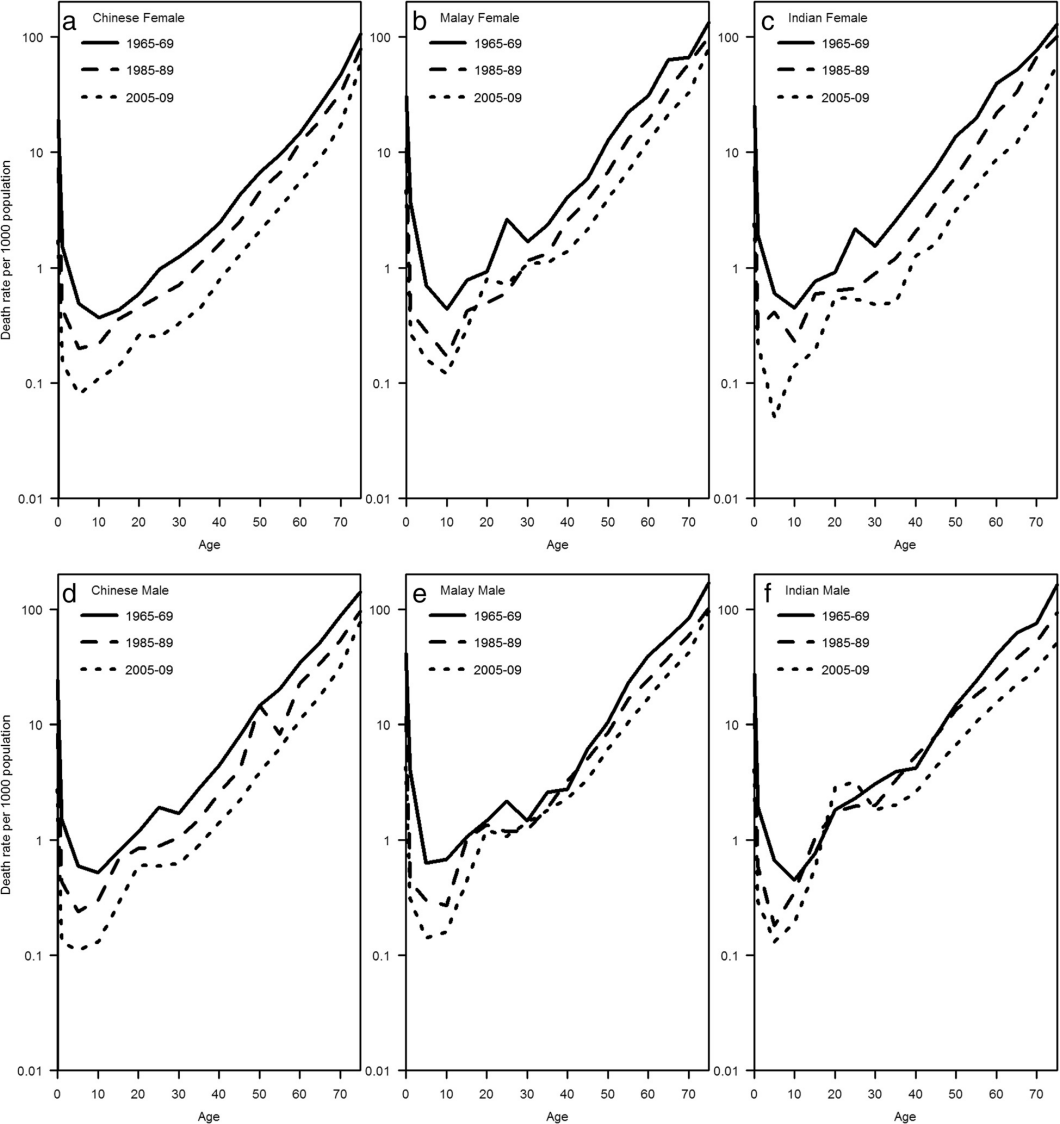
Age-specific mortality rates by ethnicity and gender (a to f), Singapore, at three time points from 1965 to 2009.

Age specific mortality rates at 3 time points are plotted in Figure 4. The graphs reflect the findings from the life expectancy calculations, and the sharpest declines in mortality rates over the 3 time points are seen in Indians (both males and females), and the smallest declines in Malays.

## Discussion

Singapore completed its epidemiological transition—from one that is heavily burdened by infectious diseases to one that is dominated by chronic, non-communicable diseases—within about 20 years (between 1950 and 1970), similar to the experience of other newly industrialising countries during the mid 20^th^ century, which also underwent the same transition over a roughly contemporaneous period [28,29]. Singaporeans have enjoyed a steady increase in life expectancy between 1965 and 2010. Males and females have similar overall life expectancy gains, accounting for the persistent gap between the genders. Although significant increases in life expectancy have occurred in all 3 ethnicities, the differential overall gains have been greatest for Indians (both genders), followed by Chinese and Malay, resulting in the convergence of life expectancy gap between the Indians and Chinese, while leaving a substantial gap between the Malays and the other two ethnic groups.

The life expectancy gap reflects differences in ethnic and gender-specific mortality rates, which might be attributable to amenable mortality (consisting of both 'treatable’ conditions such as appendicitis for which timely therapeutic care is available, and 'preventable’ conditions such as lung cancer where primary preventive measures are available) [30] leading to disparities in non-communicable disease deaths after Singapore has completed its epidemiological transition, as cancer, coronary heart disease and stroke are the 3 leading causes of mortality across the 3 ethnic groups and gender from 1970 to the present [16,18].

Cancer rates in 2006 to 2010 were highest in Chinese, followed by Malays and then Indians [31]. Indians experienced a larger decrease in terms of age standardised incidence rates for all cancers compared to the Chinese from 1968–1972 to 2003–2007 [32]. The Malays, on the other hand, experienced an increase in the age standardised incidence rates for all cancers during the same time period [32].

There are also strong ethnic differences in myocardial infarction event and case fatality rates, as well as coronary mortality rates among the 3 ethnic groups in Singapore, with higher rates in Malays and Indians than the Chinese [33,34]. The rate of decrement in crude mortality rate per 100,000 population from acute myocardial infarction was higher for Indians than for Chinese from 2007 to 2010 [34]; the Malay did not experience such a reduction [34].

Similarly, stroke rates are highest among Malays, followed by Indians and then Chinese [35]. However, the rate of decrement in crude mortality rate per 100,000 population from stroke was highest for Indians compared to the other 2 ethnic groups from 2005 to 2010 [35]. Finally, infant mortality, neonatal mortality and perinatal mortality rates were also the highest in Malays compared to the other 2 ethnic groups from 2008 to 2012 [36].

Disparities in the prevalence of risk factors for the 3 leading causes of mortality, such as tobacco use, hypertension, hyperlipidaemia and alcohol use substantially account for the differences in life expectancy within a high-income country [37]. Lifestyle factors and chronic conditions predisposing to cardiovascular disease and cancers are more prevalent in Malays: the National Health Survey (NHS) 1992 to 2010, a population representative serial cross-sectional survey [38] showed that Malays consistently have the highest prevalence of hypertension, hyperlipidaemia, smoking and obesity among the 3 ethnic groups. The National Nutrition Survey also showed that Singapore’s Malays have the highest mean saturated fat and total fat intake, as well as the lowest intake of fruits and vegetables [39]. In addition, amenable mortality was lowest for Chinese and highest for Malays, especially for treatable causes [30], suggesting there are important underlying cultural differences in health beliefs and health seeking behaviour for the 3 ethnic groups. For example, according to the NHS 2010, Malays consistently have the lowest rate of screening for colorectal, breast and cervical cancer among the 3 ethnic groups [38].

In contrast to most developed countries, we did not observe any significant convergence in life expectancy at birth, or at 65 years, between males and females in Singapore [8-10]. Again, we see substantial higher mortality rates from cancers, stroke and coronary heart disease in men compared to women. While there may be innate biological differences between the genders that explain this universal gender gap [9,10], factors that are amenable to intervention account substantially for the difference in life expectancy [37]. First, the crude prevalence of lifestyle factors and chronic conditions that predispose to cancer and cardiovascular disease is higher in men than women. The NHS showed that males consistently have a higher prevalence of smoking, hypertension, daily alcohol consumption and hyperlipidaemia than do females from 1992 to 2010. For obesity and diabetes mellitus, there was a reverse in trend for both genders. During the 1990s, women had a higher prevalence in obesity and diabetes mellitus than did men, but by 2010, the prevalence of both conditions in males had overtaken that in females.

Second, men and women in Singapore not only differ in their preferences for certain health-related habits but also in their attitudes towards health [40,41]. Survey findings from the National Health Surveillance Survey 2007 showed striking gender differences in health practices with females reporting overall better health behaviour [40,41]: they took greater conscious effort to achieve healthy nutrition such as limiting fat-intake and eating sufficient fruits and vegetables, were more likely to access health care services with the onset of mild ailments, and to have a regular family doctor whom they would consult when they had a health problem [40,41]. Of particular concern, the use of preventive medical services also differed among the elderly of both genders. Screening coverage for chronic diseases such as hypertension and hyperlipidaemia were higher in elderly women aged 60 to 69 than in men of the same age [40,41].

Third, amenable mortality for the period 1990–1994 was markedly higher in males (age standardised rate 109.7 per 100,000 population) than in females (age standardised rate 60.7 per 100,000 population) in Singapore [30]. This is consistent with the phenomenon that females in Singapore have a better health profile and are more likely to seek healthcare services in the early stage of diseases.

We do not have data about the prevalence of modifiable lifestyle risk factors prior to 1992 (except smoking). In the case of smoking, prevalence among males halved from 1970s to the present [38,42]. Lung cancer rates in males have also declined as a consequence, from 63.0 per 100,000 in the period 1978 to 1982 (age-standardised) to 40.8 per 100,000 in 2003 to 2007 [42], suggesting an impact from smoking prevalence declines. We would expect similar impacts on cardiovascular disease rates. Nevertheless, we did not notice a significantly greater gain in the rate of increase of life expectancy among males, in comparison with females. This could be due to a few reasons. Smoking is just one of many factors that could affect life expectancy, and females in Singapore have seen great improvements in some of these other factors. For example, literacy rates in women have increased from 60% in 1970 to about 96% in 2010, while the labour participation rate has also doubled from 25% in 1970 to more than 50% in 2010 [43]. Both these factors can influence health literacy and increase the level of access to healthcare services by women. There have also been significant improvements in maternal health [44].

Our data suggest that ethnic and gender health outcome inequalities persist in Singapore, and modifiable factors account for a substantial part for these differences. Culturally appropriate and sensitive evidence-based interventions organised both by government agencies (for example the Health Promotion Board) and voluntary welfare organisations targeted at males and the Malay community will be useful to reduce the life expectancy gaps that we have identified in this paper. Further research may be needed to understand the perception of risk and reasons for health-related behaviour (such as smoking), in order to develop effective interventions. Such interventions will also need to be delivered through appropriate channels that are accessed by and acceptable to the target population.

### Limitations

We highlight several limitations that may affect the interpretation of our results. First, our life expectancy measurement does not take into account the number of healthy life years lived. Health is a state of complete physical, mental and social well-being and not merely the absence of disease or infirmity, as defined by the WHO [45], and an increasing life expectancy does not necessarily mean extra years of good health. It is therefore important to take into account both quantity and quality of life by considering the use of a more suitable indicator such as *health adjusted life expectancy* (HALE), which is the number of healthy years (free from disability or disease) that a person born in a particular year can expect to live based on current trends in deaths and disease patterns [46]. The currently available data in Singapore do not allow the quantification of HALE, unfortunately.

Second, our numerator data included all mortality in Singapore, regardless of nationality status. This would have comprised deaths of non-Singapore citizens and non-Permanent Residents (PRs). The mid-year population, which formed the denominator, consisted however of only Singapore citizens and PRs. Though we are unable to remove the mortality contributed by foreign non-PRs, or to obtain the corresponding denominators including them, we believe this proportion is small as most foreign non-PRs working in Singapore are of working age when mortality is low. We do not believe that this inclusion of non-Singaporean, non-PR should affect the conclusions of our study, since the likely effect of this inclusion is to “inflate” the mortality rate in Chinese and Indians (which are large sources of non-Singaporean, non-PR persons in Singapore) relative to that of Malays. In addition, we could not differentiate between 'native-born Singaporeans’ and 'naturalised Singaporeans’ who are born outside Singapore but then become a Singapore PR or citizen in our analysis. However our objective in this paper is to evaluate changes in life expectancy of the Singapore population over time regardless of their place of birth, and as such we do not view this distinction as critical.

Third, period life expectancy is used in this study rather than cohort life expectancy. Period life expectancy can be interpreted as the mean age at death that is experienced by a real cohort under the assumption that age-specific mortality rates observed during that period apply to that cohort [47]. Cohort life expectancy, on the other hand, summarises the mortality experience of an actual birth cohort of individuals as they age over time, from birth until the cohort becomes extinct through the death of the last survivor [45]. Though it is technically easier and faster to report period life expectancy, the limitation is that period life expectancy will not reflect cohort changes in life expectancy accurately in the case that age-specific mortality rates are changing over time [48]. This was observed when there was a sudden peak in the life expectancy among Indian males around year 2000. We believe that one of the reasons for this sudden peak might be the surge of PRs in the Indian male resident population. The proportion of Indian male PRs doubled from 9.5% in 1990 to 18.0% in 2000, which corresponds to the largest increment among the ethnic-gender combinations in Singapore for that period. This 'healthy migrant’ effect where immigrants are on average healthier than the native-born [49] can cause a sudden change in mortality rates and life expectancy, and the use of period life expectancy will not be able to reflect this well.

## Conclusions

In summary, we find ethnic and gender specific differences in life expectancy at birth and at 65 years in Singapore that have persisted between 1965 and 2009 due to differential rates in life expectancy gain. Modifiable factors account for a substantial part for these differences. Health promotion activities targeted at at-risk communities (males, Malays) to address these modifiable factors could reduce these differences.

## Appendix I: formulae used in the computation process of life expectancy from 1965 to 2009

### Age-specific mortality rate

The age-specific mortality rate _*n*_*M*_*x*_ can be computed using _*n*_*D*_*x*,_ the number of deaths occurring to persons aged *x* to *x + n* throughout the 5-year period, and _*n*_*P*_*x,*_ the number of persons years lived by persons aged *x* to *x + n* throughout the 5-year period [22-24]. Since each length of period under consideration is 5 years, we multiply the number of persons aged *x* to *x + n* alive at the mid-point of the period by five to get _*n*_*P*_*x*_. For most age groups, *n = 5* as the population is categorized into 5-year age groups, except for the youngest two age groups (< 1 and 1 **–** 4) and the oldest age group (75+),

$${}_{n}M_{x}{=}_{n}D_{x}{/}_{n}P_{x}.$$

### Probability of dying between age x and x + n

Assuming that deaths are spread evenly across the 5-year period, the probability of dying, _*n*_*q*_*x,,*_ for the general age groups is

$${}_{n}q_{x}=2\cdot n\cdot_{n}M_{x}/\left(2+n\cdot_{n}M_{x}\right).$$

However, for infants, deaths tend to be concentrated at the first few months of life. Hence, the assumption that deaths are spread evenly across the observed period does not hold. Considering this factor, the infant mortality rate is used instead of the above equation. For persons aged 0 to 1, we have

_1_*q*_0_  =  _1_*D*_0_ /*B*, where *B =* number of live births over the 5-year period.

For the open-ended oldest age group, _*∞*_*q*_*x*_ *= 1* as everyone in this age group will eventually die without entering another age group [22-24].

### Number of survivors at age x

The radix of the life table, *l*_*0*_, is set at 100,000. The number of persons expected to be alive at age *x, l*_*x*_*,* is derived based on the formula

$$l_{x}=l_{x-n}–\left(l_{x-n}\cdot_{n}q_{x-n}\right).$$

### Number of deaths between age x and x + n

The number of persons expected to die between age *x* to *x + n*, _*n*_*d*_*x*_, is obtained by multiplying the number of survivors at age *x, l*_*x*_, by the probability of dying between age *x* to *x + n*, _*n*_*q*_*x.*_ That is,

$${}_{n}d_{x}=l_{x}\cdot_{n}q_{x}.$$

### Number of person-years lived between age x and x + n

For non-infants, assuming that deaths are uniformly distributed within the age interval and across the 5-year period, _*n*_*L*_*x*_, the number of person-years lived within age *x* and *x + n* by survivors at age *x* is computed as

$${}_{n}L_{x}=\left(n/2\right)\cdot \left(l_{x}+l_{x+n}\right).$$

For infants, the above formula is modified using separation factors to allocate different weights to *l*_*0*_ and *l*_*1.*_ As the majority of deaths occur within the first few months of life, a smaller weight of 0.3 is given to *l*_*0*,_ while a larger weight of 0.7 is given to *l*_*1*._ These values are adapted from Coale-Demeny equations [Coale A. J. and P. Demeny, 1983, Regional Model Life Tables and Stable Populations, 2^nd^ edition, Academic Press, New York]. The number of person-years lived within age 0 and 1 by survivors at age 0 is computed as

$${}_{1}L_{0}=0.3\left(l_{0}\right)+0.7\left(l_{1}\right).$$

For the open-ended oldest age group, the number of persons dying in the period above age *x* must be equal to the number of persons surviving to age *x (*_*∞*_*d*_*x*_ *= l*_*x*_*)*, so

$$\infty Lx=l_{x}/{(}_{\infty }M_{x}).$$

### Total person-years lived above age x

The total number of person-years expected to be lived by survivors at age *x*, *T*_*x,*_ is calculated by summing the number of person-years lived, _*n*_*L*_*x*,_ from the oldest to the youngest age [22-24].

$$\text{Tx}=\sum_{\alpha =x}^{\propto }{}_{n}L_{a}$$

### Life expectancy at age x

The average number of years which a person alive at age *x* is expected to live, *e*_*x*,_ is obtained by dividing the total number of person-years expected to be lived at age *x* by the number of survivors at age *x*[22-24].

$$e_{x}=\left(T_{x}\right)/\left(l_{x}\right).$$

## Abbreviations

HALE: Health adjusted life expectancy; ICD: International classification of diseases; NHS: National health survey; PRs: Permanent residents; WHO: World health organisation.

## Competing interests

The authors declare that they have no competing interests.

## Authors’ contributions

RBTL interpreted the data, developed the tables, drafted and revised the manuscript. HZ analysed the data, developed the graphs and helped revised the manuscript. QY analysed the data and assisted to develop the graphs. ARC revised the manuscript critically for important intellectual content, and provided guidance to improve the graphs. KSC was involved in the conception and design of the study, and revised the manuscript critically for important intellectual content. WYL was involved in the conception and design of the study, interpreted the data, and revised the manuscript critically for important intellectual content. All authors read and approve the final manuscript.

## Pre-publication history

The pre-publication history for this paper can be accessed here:

http://www.biomedcentral.com/1471-2458/13/1012/prepub
